# Clinicopathological Features of DLL3 Expression and T‐Cell Infiltration in LCNEC: Insights From Pathology and Clinical Response to Tarlatamab

**DOI:** 10.1111/1759-7714.70400

**Published:** 2026-10-15

**Authors:** Shotaro Kaneko, Naoki Furuya, Yoshihisa Sugiura, Hajime Tsuruoka, Kazuhiro Nishiyama, Honoka Morita, Tomoki Okura, Hisashi Saji, Junki Koike, Nobuyuki Ohike, Masamichi Mineshita

**Affiliations:** ^1^ Department of Respiratory Medicine St. Marianna University School of Medicine Kawasaki, Kanagawa Japan; ^2^ Division of Molecular Pathology, Department of Pathology St. Marianna University School of Medicine Kawasaki, Kanagawa Japan; ^3^ Department of Thoracic Surgery St. Marianna University School of Medicine Kawasaki, Kanagawa Japan; ^4^ Department of Pathology St. Marianna University School of Medicine Kawasaki, Kanagawa Japan

**Keywords:** bispecific T‐cell engager, CD8, DLL3, tarlatamab, T‐cell infiltration

## Abstract

**Background:**

Large cell neuroendocrine carcinoma (LCNEC) is a rare and aggressive lung neuroendocrine neoplasm with poor prognosis. Recently, DLL3‐targeted bispecific T‐cell engagers (BiTEs) have shown promising efficacy in small cell lung cancer, and clinical trials are ongoing in LCNEC. This study evaluated DLL3 expression and CD8+ tumor‐infiltrating lymphocytes (TILs) infiltration in LCNEC and investigated their clinical significance and association with tarlatamab efficacy.

**Methods:**

We retrospectively reviewed consecutive patients who were diagnosed with LCNEC. Immunohistochemical analyses for DLL3 and CD8 were performed. DLL3 positivity was defined as staining in ≥ 1% of tumor cells. CD8+ TIL infiltration was graded as low, moderate, or high.

**Results:**

Among 77 screened LCNEC cases, 44 were eligible for analysis. The median age was 75 years. DLL3 expression was observed in 70.5% of cases and was associated with worse survival. Low CD8+ TIL infiltration was observed in 59.1% of cases. Patients with high CD8+ TIL infiltration demonstrated significantly longer survival than those with low infiltration (*p* = 0.03). Among representative clinical cases, a patient with DLL3 (+)/CD8 TILs (−) LCNEC achieved marked tumor shrinkage, whereas a patient with DLL3 (−)/CD8 TILs (−) LCNEC showed no response.

**Conclusions:**

DLL3 is frequently expressed in LCNEC and correlates with a poorer prognosis. A majority of LCNEC cases are immunologically characterized as cold tumors lacking CD8+ TILs, which contributes to unfavorable clinical outcomes. Tarlatamab induced a robust antitumor response even in a DLL3 (+) cold tumor. DLL3‐BiTE represents a promising therapeutic strategy for DLL3 (+) LCNEC.

## Graphical abstract

This study suggested that DLL3 is frequently expressed in LCNEC and correlates with a poor prognosis. A majority of LCNEC cases are immunologically characterized as cold tumors lacking CD8+ TILs, which contributes to unfavorable clinical outcomes.

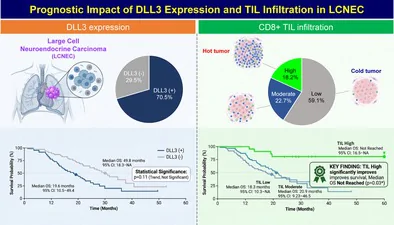

## 1 Introduction

Large cell neuroendocrine carcinoma (LCNEC) is classified as one of the lung neuroendocrine neoplasms and is characterized by aggressive tumor progression with poor survival rates, similar to small cell lung cancer (SCLC) [1]. LCNEC is a relatively rare histological subtype, accounting for only 3% of all lung cancers [2]. Diagnostic criteria for LCNEC include neuroendocrine morphology (trabeculae, palisading, organoid nesting, and rosette formation), a high proliferation rate, extensive geographical necrosis, and immunohistochemical expression of at least one neuroendocrine marker (chromogranin A, synaptophysin, and CD56). Features that differentiate LCNEC from SCLC include large cells with prominent eosinophilic cytoplasm, numerous prominent nucleoli, and a low nuclear‐to‐cytoplasmic ratio [3, 4]. Since LCNEC is a rare histological subtype, there are no established treatments based on the results of Phase III studies. Therefore, most patients with advanced‐stage LCNEC are treated with combination therapy including immune checkpoint inhibitors (ICIs) or platinum doublet chemotherapy, similar to the approach used for SCLC in clinical practice [5].

Delta‐like ligand 3 (DLL3) is a member of the Notch ligand family that functions as an inhibitory ligand of the Notch receptor. DLL3 is predominantly expressed on the surface of various neuroendocrine carcinomas (NECs) but exhibits minimal to no expression in normal adult tissues [6, 7]. The DLL3‐Notch axis plays a critical role in cancer development, proliferation, invasion, and metastasis in NECs. DLL3 is aberrantly expressed on the tumor cell surface in 85%–94% of patients with SCLC, making DLL3 an attractive therapeutic target [8, 9, 10, 11]. Consequently, various DLL3‐targeted therapies have been under development for SCLC and other NECs [12, 13].

Tarlatamab, a bispecific antibody targeting both DLL3 and the T cell surface antigen CD3, was developed as a bispecific T‐cell engager (BiTE) and demonstrated promising durable antitumor activity in a Phase I/II study for advanced‐stage SCLC [14, 15]. In a recent Phase III study, tarlatamab significantly prolonged both overall survival (OS) and progression‐free survival (PFS) compared with standard single‐agent chemotherapy [16], Consequently, tarlatamab has been established as a standard treatment for advanced‐stage SCLC after failure of platinum‐doublet chemotherapy with or without ICIs. Tarlatamab exerts its antitumor effect by bridging DLL3‐expressing tumor cells with CD3+ T‐cells, predominantly CD8+ cytotoxic T‐cells, thereby inducing an antitumor immune response [17]. Therefore, the therapeutic efficacy of DLL3‐targeted BiTE therapy may depend on the presence and functional status of CD8+ tumor‐infiltrating lymphocytes (CD8+ TILs) within the tumor microenvironment (TME) [6]. Previous studies suggested that high CD8+ TILs infiltration was a positive predictive biomarker for the efficacy of ICIs in nonsmall cell lung cancer (NSCLC), and tumors with high CD8+ TILs are often referred to as “hot tumors” [18]. In contrast, SCLC is characterized by low CD8+ TILs infiltration, and this immunosuppressive TME is referred to as “cold tumors,” which have been implicated in the limited responsiveness to ICIs [19, 20].

Given the immunohistochemical and biological similarities between LCNEC and SCLC, DLL3 overexpression and low CD8+ TILs infiltration have also been suggested to be characteristic features of LCNEC. Based on these similarities to SCLC, several early‐phase clinical trials investigating the efficacy of other T‐cell engagers in LCNEC are currently ongoing [13, 21]. However, previous studies have reported inconsistent rates of DLL3 expression, ranging from 37% to 78% [22, 23, 24, 25, 26, 27], likely because of differences in the DLL3 antibodies used for IHC and the cut‐off values adopted across studies. Furthermore, simultaneous evaluation of CD8+ TILs infiltration and DLL3 expression may help to predict the therapeutic efficacy of DLL3‐BiTE in LCNEC. The objectives of this study are to evaluate DLL3 expression and CD8+ TILs infiltration in LCNEC and to investigate their clinical significance. In addition, this study aims to examine correlations between these pathological findings and the therapeutic efficacy of tarlatamab in patients with LCNEC.

## 2 Methods

### 2.1 Patients

We retrospectively reviewed consecutive patients who were diagnosed with LCNEC pathologically between January 2015 and September 2025 at St. Marianna University Hospital. Patients were excluded if they met any of the following criteria: (i) cases in which the final diagnosis was not confirmed as LCNEC, (ii) specimens were deemed inadequate for immunohistochemical analysis due to insufficient tissue or poor quality. Clinical data were obtained from medical records according to study protocol. The clinical features included age at diagnosis, sex, smoking history, clinical/pathological stage at diagnosis (UICC 9th edition), and history of chemotherapy. The data cut‐off date was February 28, 2026. This study was approved by the institutional review board of St. Marianna University School of Medicine (approval no. 6652).

### 2.2 Histological Evaluation

To ensure diagnostic accuracy of LCNEC, all histological evaluations were independently re‐evaluated by two board‐certified pathologists. The diagnosis of LCNEC was confirmed based on the criteria of the 2021 World Health Organization (WHO) classification of lung tumors [3]. The pathological confirmation of LCNEC used hematoxylin and eosin (H&E) staining and neuroendocrine markers by immunohistochemistry (IHC) including synaptophysin and CD56. Both pure LCNEC and combined LCNEC were included in this study. We evaluated DLL3 expression and CD8+ TILs in the LCNEC component only.

### 2.3 Immunohistochemistry

We prepared 4 μm thick sections from formalin‐fixed paraffin‐embedded (FFPE) tissue blocks mounted on adhesive‐coated glass slides (CRE‐11; Matsunami Glass Ind. Ltd., Osaka, Japan) for DLL3 and CD8. IHC staining was performed using an automated stainer (Histostainer 48A; Nichirei Biosciences Inc., Tokyo, Japan). Immunoreactivity was visualized with 3,3′‐diaminobenzidine (DAB) (Liquid DAB+ Substrate Chromogen System; Dako/Agilent Technologies, Santa Clara, CA, USA; K3468).

For DLL3 staining, antigen retrieval was carried out in Tris/EDTA buffer (Epitope Retrieval Solution, pH 9; RE7119, Leica Biosystems, Nussloch, Germany) at 98°C for 88 min, followed by gradual cooling at room temperature for 20 min. Endogenous peroxidase activity was blocked for 4 min using the peroxidase inhibitor included in the OptiView DAB Universal Kit (Ventana Medical Systems/Roche Diagnostics, Tucson, AZ, USA). Sections were incubated with a primary anti‐DLL3 rabbit monoclonal antibody (clone SP347; Ventana/Roche; ready‐to‐use) for 32 min. Signal detection was performed using the HQ Linker and HRP Multimer from the same kit, each applied for 8 min. The DAB chromogenic reaction was carried out for 4 min, followed by copper enhancement for an additional 4 min. DLL3 positivity was confirmed when ≥ 1% of tumor cells showed staining and negative for < 1%, according to previous studies [14, 15, 16].

For CD8 staining, antigen retrieval was performed in citrate buffer (pH 6.0; RM102‐C, LSI Medience, Tokyo, Japan) at 98°C for 40 min, followed by gradual cooling at room temperature for 20 min. Endogenous peroxidase activity was blocked with 3% hydrogen peroxide for 5 min. Sections were then incubated with a primary anti‐CD8 mouse monoclonal antibody (clone C8/144B; Dako/Agilent; diluted 1:100) for 90 min. Detection was performed using a polymer‐based method (Histofine Simple Stain MAX‐PO MULTI; Nichirei Biosciences; code 724152) for 30 min. The DAB reaction was carried out for 5 min. CD8+ cells were graded based on the number of positive cells per high‐power field (HPF). The scoring system of CD8+ TILs density was defined as follows: low, < 30 cells/HPF; moderate, 30–69 cells/HPF; and high, ≥ 70 cells/HPF, according to previous studies [18, 28]. DLL3 and CD8 IHC results were evaluated by two board‐certified pathologists at our institution, who were blinded to all clinical and other pathological data.

### 2.4 Statistical Analysis

All statistical analyses were performed using EZR version 1.68 (Saitama Medical Center, Jichi Medical University, Saitama, Japan) [29], which is a graphical user interface for R version 4.3.1 (The R Foundation for Statistical Computing, Vienna, Austria). Survival curves were calculated by Kaplan–Meier method and compared by log‐rank test according to DLL3 expression and CD8+ TILs infiltration grade. To compare survival outcomes among the three groups based on TIL grade (low, moderate, and high), pairwise comparisons were performed, and *p*‐values were adjusted using the Holm method to control the family‐wise error rate. Categorical variables were compared using Fisher's exact test. Continuous variables were analyzed using the Mann–Whitney U test for comparisons between two groups, and the Kruskal‐Wallis test for comparisons among the three groups. All *p*‐values were two‐sided, and a *p*‐value of < 0.05 was considered statistically significant.

## 3 Results

### 3.1 Patient Characteristics

We initially screened 77 cases with a diagnosis or suspicion of LCNEC from pathology reports. After re‐evaluation of LCNEC, a total of 33 cases were excluded: 24 due to an inconclusive diagnosis of LCNEC and 9 due to the unavailability of specimens for IHC. A total of 44 cases were identified as a final analysis set (Figure 1). Patient characteristics are summarized in Table 1. The median age at diagnosis was 75 years old. Male and smokers were dominant. Pathological specimens were obtained via biopsy in 16 cases (36.4%) and surgical resection in 28 cases (63.6%). The disease stage at diagnosis as follows: Stage I (*n* = 22, 50.0%), Stage II (*n* = 4, 9.1%), Stage III (*n* = 6, 13.6%), and Stage IV (*n* = 12, 27.3%). Regarding histological subtypes, 13 cases (29.5%) were identified as pure LCNEC and 31 cases (70.5%) as combined LCNEC. The details of systemic treatment regimens and the corresponding numbers of patients are summarized in Table S1.

**FIGURE 1:**
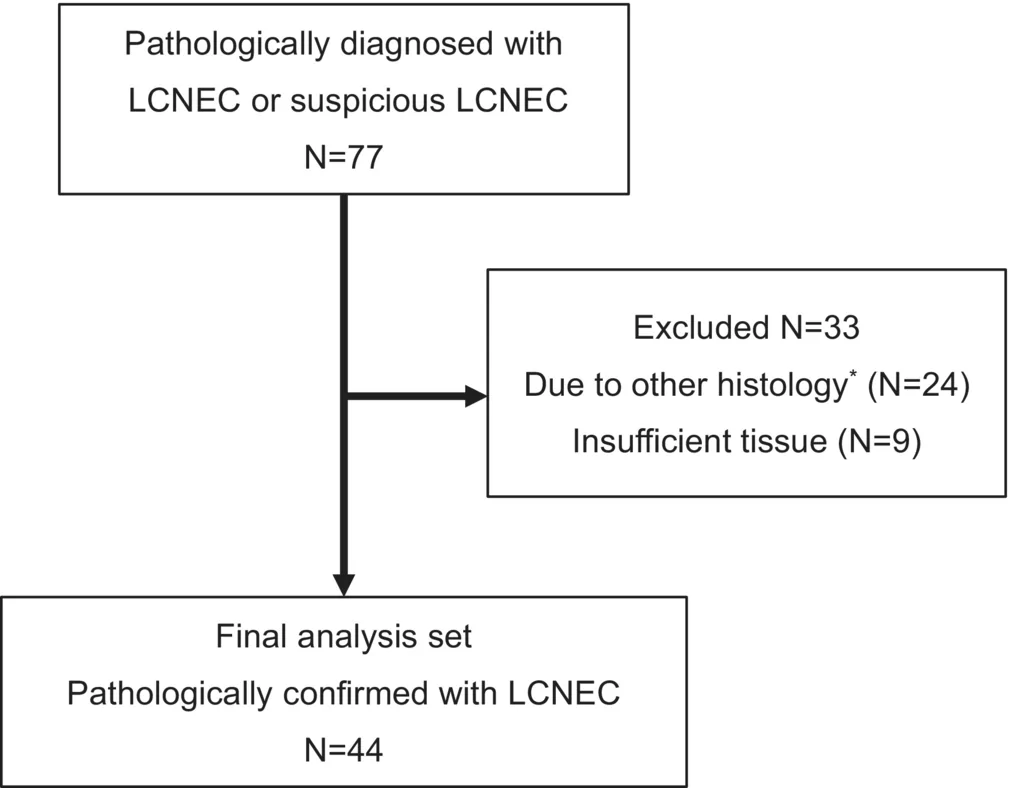
Consort diagram of the study. Twenty‐four patients were excluded due to other histology (15 small cell carcinoma, 4 not otherwise specified (NOS), 2 adenocarcinoma, 1 squamous cell carcinoma, 1 large cell carcinoma (non‐NEC), 1 SMARCA4 deficient carcinoma).

**TABLE 1:** Patient characteristics (*N* = 44).

	Number of patients (%)
Median age (range)	75 (51–84)
Sex, male/female	36 (81.8%)/8 (18.2%)
Smoking history, (+)/(−)	43 (97.7%)/1 (2.3%)
PS at diagnosis, 0/1/2/3/4/unknown	28 (63.6%)/10 (22.7%)/2 (4.6%)/1 (2.3%)/1 (2.3%)/2 (4.6%)
Specimens, biopsy/surgical resection	16 (36.4%)/28 (63.6%)
Sample, primary lesion/metastatic site	40 (90.9%)/4 (9.1%)
Stage at diagnosis, I/II/III/IV	22 (50.0%)/4 (9.1%)/6 (13.6%)/12 (27.3%)
Histology, pure LCNEC/combined LCNEC	13 (29.5%)/31 (70.5%)
Initial Treatment, Curative treatment^a^/Palliative chemotherapy	30 (68.2%)/14 (31.8%)

^a^ Curative treatments included surgical resection alone (*N* = 17), surgical resection followed by adjuvant chemotherapy (*N* = 10) and definitive chemoradiotherapy (*N* = 2). Of these patients, 12 patients were treated with palliative chemotherapy as post curative treatment reccurence.

### 3.2 DLL3 Expression and CD8+ TILs Infiltration

Representative examples of DLL3‐negative/positive tumors and density of CD8 + TILs are shown in Figure 2. DLL3 expression was positive in 31 cases (70.5%) and negative in 13 cases (29.5%), using a cut‐off of ≥ 1% positive tumor cells (Figure 3A). CD8+ TILs infiltration grade low, moderate, and high were observed in 26 (59.1%), 10 (22.7%), and 8 (18.2%) cases, respectively (Figure 3B). Most patients with LCNEC exhibited an immune‐cold phenotype within the TME. No statistically significant differences in clinical features were observed between the DLL3 (+) and DLL3 (−) populations (Table 2). Similarly, there were no significant differences in clinical characteristics among the groups stratified by CD8+ TILs infiltration grade (Table 3). To evaluate the relationship between DLL3 expression and CD8+ TIL density, we provided a box‐and‐whisker plot (Supplementary Fig. S1). Although the median number of CD8+ TILs was lower in the DLL3 (+) population than in the DLL3 (−) population (5 cells/HPF vs. 35 cells/HPF, *p* = 0.34), the difference was not statistically significant.

**FIGURE 2:**
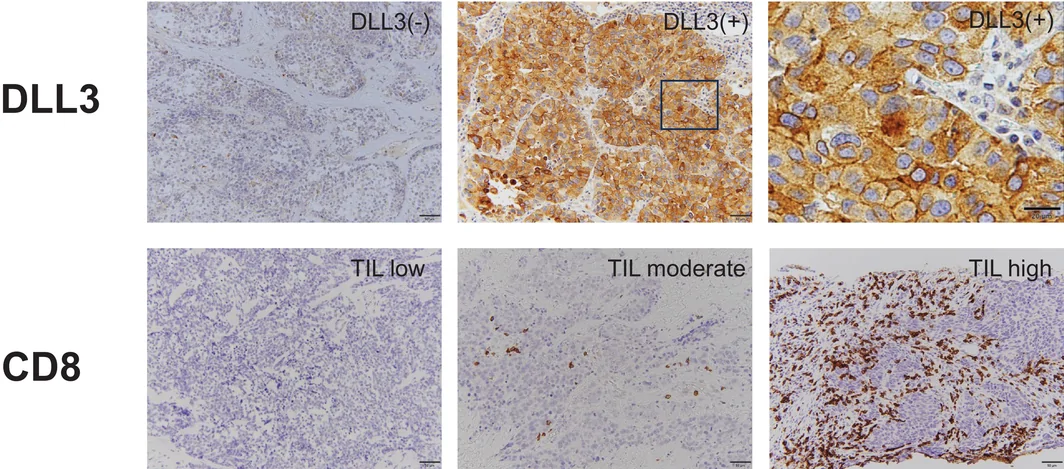
Representative immunohistochemistry of DLL3 and CD8+ TILs. Representative examples of DLL3‐negative/positive tumors and density of CD8 + TILs. TILs: Tumor‐infiltrating lymphocytes.

**FIGURE 3:**
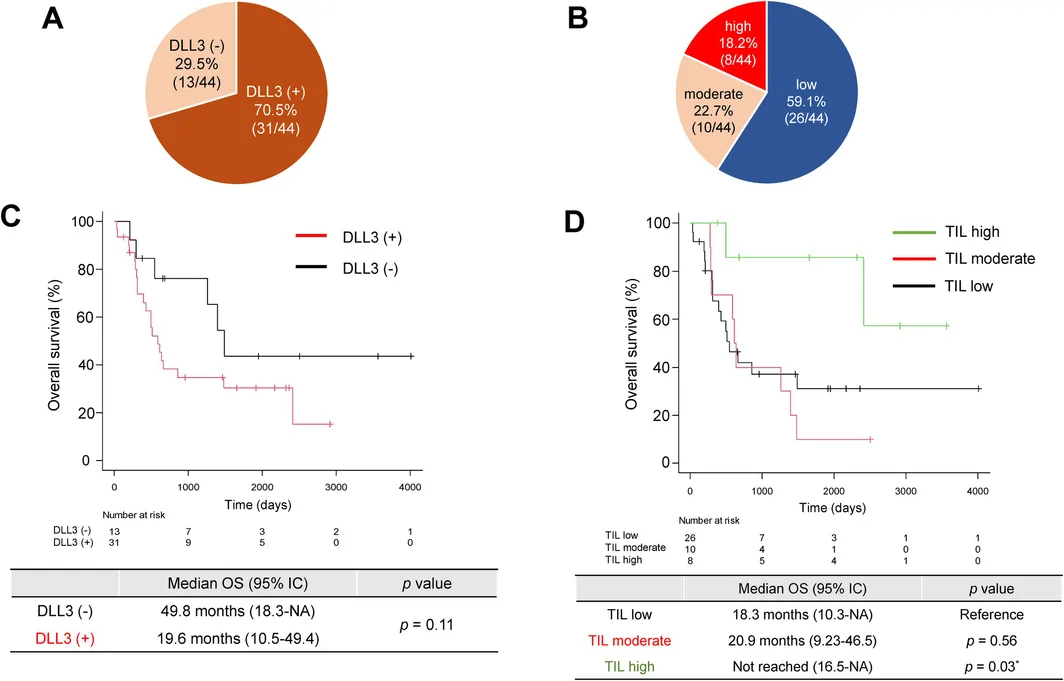
Prevalence of DLL3 expression and CD8+ TILs infiltration, and survival outcomes. (A) DLL3 expression was positive in 31 cases (70.5%) and negative in 13 cases (29.5%), using a cut‐off of ≥ 1% positive tumor cells. (B) CD8+ TILs infiltration grade of low, moderate, and high was observed in 26 (59.1%), 10 (22.7%), and 8 (18.2%) cases, respectively. (C) Survival outcomes according to DLL3 expression. The median OS was 49.8 months in the DLL3 (−) population and 19.6 months in the DLL3 (+) population. (D) Survival outcomes according to CD8+ TILs infiltration. High TILs grade population had significantly longer OS compared to low TILs grade population (median OS not reached vs. 18.3 months, *p* = 0.03).

**TABLE 2:** Patient characteristics according to DLL3 expression.

	Number of patients (%), DLL3 (−) (*N* = 13)	Number of patients (%), DLL3 (+) (*N* = 31)	*p*
Median age (range)	75 (54–83)	75 (69–84)	*p* = 0.806
Sex, male/female	10 (76.9%)/3 (23.1%)	26 (83.9%)/5 (16.1%)	*p* = 0.676
Smoking history, (+)/(−)	13 (100%)/0 (0%)	30 (96.8%)/1 (3.2%)	*p* = 1
Stage at diagnosis I–II/III–IV	9 (69.2%)/4 (30.8%)	17 (54.8%)/14 (45.2%)	*p* = 0.507
Histology, pure LCNEC/combined LCNEC	10 (76.9%)/3 (23.1%)	21 (67.7%)/10 (32.3%)	*p* = 0.722
Anti‐PD‐L1 antibody treatment, (+)/(−)	3 (6.8%)/10 (22.7%)	9 (20.5%)/22 (50.0%)	*p* = 1

**TABLE 3:** Patient characteristics according to TILs grade.

	Number of patients (%), Low (*N* = 26)	Number of patients (%), Moderate (*N* = 10)	Number of patients (%), High (*N* = 8)	*p*
Median age (range)	75 (51–84)	78 (69–83)	74 (58–79)	*p* = 0.173
Sex, male/female	20 (76.9%)/6 (23.1%)	9 (90%)/1 (10%)	1 (12.5%)/7 (87.5%)	*p* = 0.76
Smoking history, (+)/(−)	25 (96.2%)/1 (3.8%)	10 (100%)/0 (0%)	8 (100%)/0 (0%)	*p* = 1
Stage at diagnosis, I–II/III–IV	13 (50%)/13 (50%)	8 (80%)/2 (20%)	5 (62.5%)/3 (37.5%)	*p* = 0.275
Histology, pure LCNEC/combined LCNEC	18 (69.2%)/8 (30.8%)	7 (70%)/3 (30%)	6 (75%)/2 (25%)	*p* = 1
Anti‐PD‐L1 antibody treatment, (+)/(−)	7 (15.9%)/19 (43.2%)	2 (4.5%)/8 (18.2%)	3 (6.8%)/5 (11.4%)	*p* = 0.802

### 3.3 Prognostic Impacts of DLL3 Expression and CD8+ TILs Infiltration

The prognostic impact of DLL3 expression and CD8+ TILs infiltration on overall survival (OS) was evaluated from initial diagnosis to death. The median follow‐up was 627 days (20.9 months). Regarding DLL3 expression, the median OS was 49.8 months in the DLL3 (−) population and 19.6 months in the DLL3 (+) population. DLL3 (+) showed worse OS, though not significantly different (Figure 3C). In contrast, regarding CD8+ TILs infiltration, the high TILs grade population had a significantly longer OS compared to the low TILs grade population (median OS not reached vs. 18.3 months, *p* = 0.03, Figure 3D). Figures S2 and S3 show the results of subgroup analyses evaluating the prognostic impact of DLL3 expression and CD8+ TILs infiltration in two separate initial treatment subgroups: the curative treatment population and the palliative chemotherapy population.

Furthermore, we performed an exploratory analysis by dividing the cohort into four groups based on DLL3 expression and CD8+ TILs status: DLL3 (+)/CD8 TILs (+), DLL3 (+)/CD8 TILs (−), DLL3 (−)/CD8 TILs (+), and DLL3 (−)/CD8 TILs (−). Notably, the DLL3 (+)/CD8 TILs (−) group exhibited the shortest median OS (16.4 months) among all phenotypes (Figure 4).

**FIGURE 4:**
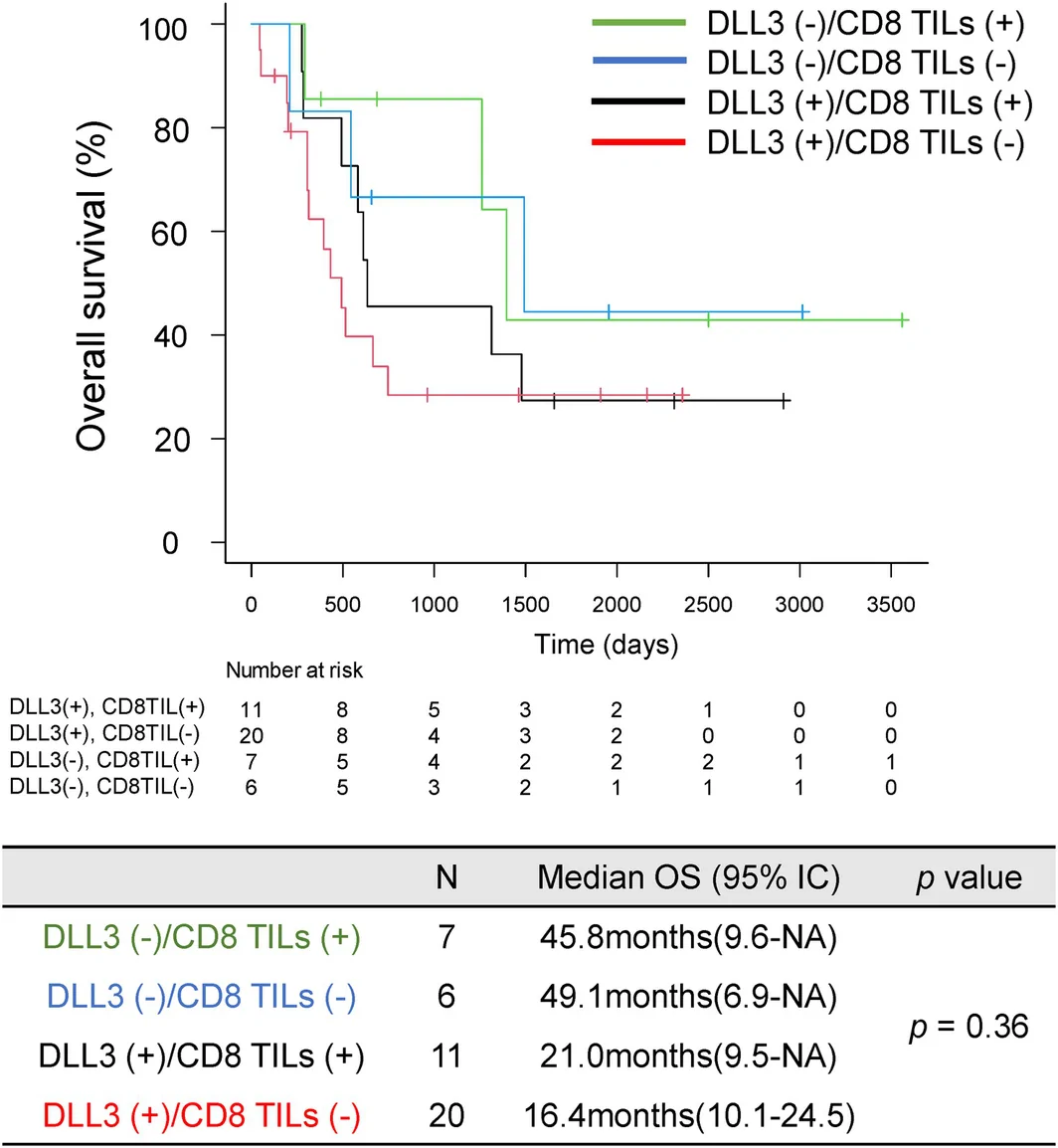
Survival outcomes according to DLL3 expression and CD8+ TILs infiltration. Kaplan–Meier curves for overall survival (OS) stratified by the combination of DLL3 expression and CD8+ tumor‐infiltrating lymphocytes (TILs) status. The DLL3(+)/CD8(−) population (red line) exhibited the shortest median OS (16.4 months) among all phenotypes.

### 3.4 Representative Cases

#### 3.4.1 Case 1: DLL3 (+)/CD8 TILs (−)

A 73‐year‐old female with a heavy smoking history presented with a large mediastinal tumor involving airway invasion and stenosis. Pathological specimens were obtained via transbronchial biopsy, confirming a diagnosis of LCNEC, based on morphological findings, positivity of CD56, and synaptophysin. The clinical stage was IVB (cT3N3M1b) with bone metastases. As first‐line treatment, the patient received combination therapy with carboplatin, etoposide, and atezolizumab. After four cycles of induction therapy, the initial best response was partial response (PR). However, during subsequent maintenance therapy with atezolizumab, the subcarinal mediastinal lymph node enlarged, leading to dyspnea and cough due to left main bronchial stenosis. IHC revealed that the tumor was DLL3 (+) but negative for CD8+ TILs infiltration. Tarlatamab was immediately administrated as second‐line treatment. Two months after the initiation of tarlatamab, the subcarinal mediastinal lymph node rapidly shrunk, and the patient's symptoms improved (Figure 5A). This patient continued to receive tarlatamab for over 6 months.

**FIGURE 5:**
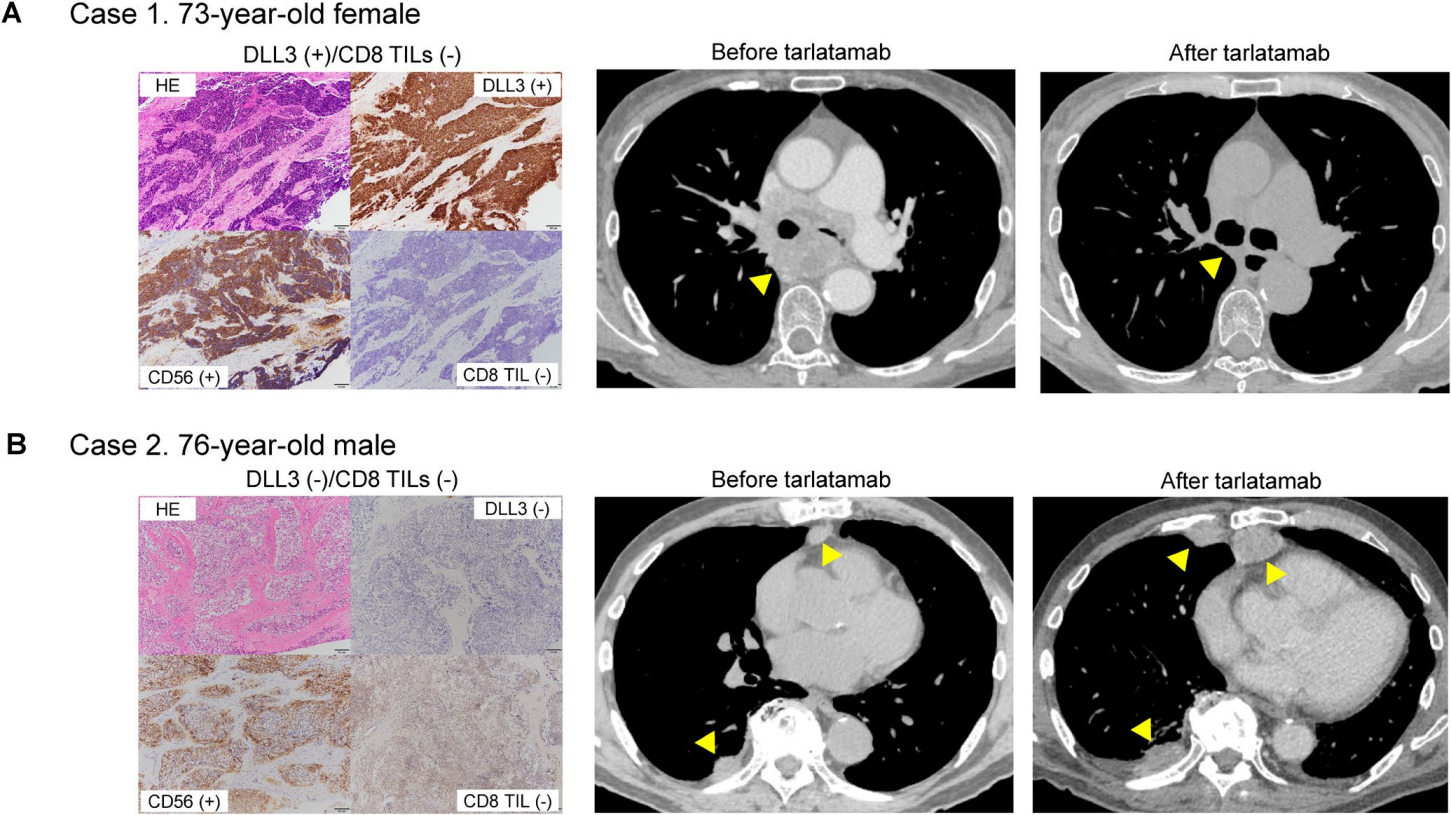
Representative Cases treated with tarlatamab. (A) LCNEC case with DLL3 (+)/CD8 TILs (−). Two months after the initiation of tarlatamab, the subcarinal mediastinal lymph node had rapidly shrunk (arrows), and the patient's symptoms improved. (B) LCNEC case with DLL3 (−)/CD8 TILs (−). Two months after initiation of tarlatamab, pleural disseminations were further enlarged (arrows).

#### 3.4.2 Case 2: DLL3 (−)/CD8 TILs (−)

A 76‐year‐old male with a significant smoking history received complete resection of a mediastinal tumor, which was pathologically confirmed as LCNEC with positive expression for both CD56 and synaptophysin. The pathological stage was IIIA (pT3N2aM0). Fifteen months after radical surgery, bone metastases and pleural dissemination were documented. As first‐line treatment, the patient received combination therapy consisting of carboplatin, etoposide, and atezolizumab. However, after two cycles, both the bone metastases and pleural dissemination had progressed. IHC revealed that the tumor was DLL3 (−) and showed no CD8+ TILs infiltration. Subsequently, tarlatamab was initiated as a second‐line treatment. Despite this, 2 months after initiation of tarlatamab, the bone metastases and pleural dissemination were further enlarged. As tarlatamab failed to demonstrate any antitumor efficacy, the patient's performance status deteriorated from PS 1 to PS 3 (Figure 5B).

## 4 Discussion

We evaluated DLL3 expression and CD8+ TILs infiltration in LCNEC by IHC and investigated their prognostic impact. DLL3 expression was observed in 70.5% of LCNEC cases, and the DLL3 (+) population had worse survival outcomes compared to those in the DLL3 (−) population. Around 60% of LCNEC cases were cold tumors which were negative for CD8+ TILs infiltration and had significantly worse OS. Furthermore, we experienced two LCNEC cases which were treated with tarlatamab. One DLL3 (+) case demonstrated a remarkable response to tarlatamab despite CD8+ TILs infiltration. In contrast, tarlatamab failed to demonstrate any antitumor efficacy in the DLL3 (−) case.

To compare the positivity of DLL3 expression with SCLC, DLL3 antibody SP347 and a cut‐off value ≥ 1% positive tumor cells were adopted from previous studies [14, 15, 16]. In our cohort, 70.5% positivity was comparable to previous studies [22, 23, 24, 25]; however, the prevalence of DLL3 positivity for LCNEC was relatively lower than SCLC [8, 9, 10, 11]. LCNEC is a heterogeneous population with different genetic profiles depending on the subtype (SCLC‐like, NSCLC‐like, or Carcinoid‐like) [1], which might influence DLL3 expression. Regarding the prognostic impact of DLL3 expression, the median OS of DLL3 (+) population was worse compared to DLL3 (−) populations, which was consistent with a previous report [26]. This may suggest that biological characteristics of DLL3 are associated with increased aggressiveness and proliferation in neuroendocrine neoplasms [1, 6].

High‐grade pulmonary neuroendocrine tumors, including LCNEC, are generally characterized by low immune cell infiltration within TME and are often referred to as cold tumors [30, 31]. In the present study, 59.1% of LCNEC were low TILs grade, reflecting a paucity of lymphocyte infiltration. Low TILs infiltration indicates that an anti‐tumor immune response is suppressive and contributes to worsening survival, consistent with previous studies for NSCLC [32, 33] and LCNEC [34]. Cold tumors are also associated with a poor prognosis and diminished efficacy of ICIs [34]. Recently, Shirasawa et al. [35] demonstrated that DLL3‐high SCLC exhibit profound immunosuppression driven by the downregulation of antigen‐presentation machinery (e.g., dendritic cell functions and the STING pathway) and impaired chemokine‐mediated T‐cell trafficking. Consequently, despite harboring high neoantigen loads, DLL3‐high tumors develop an immune‐cold phenotype depleted of TILs, rendering them resistant to anti‐PD‐L1 chemo‐immunotherapy [35]. Consistent with these findings, our data revealed that patients with DLL3 (+)/CD8 TILs (−) tumors exhibited worse clinical outcomes compared to other phenotypes (Figure 4). Together, our results reinforce the notion that DLL3‐mediated suppression of T‐cell infiltration contributes to an immune‐evasive environment and poor prognosis, aligning with the mechanistic model proposed by Shirasawa et al.

While tarlatamab has been approved as a standard of care for pretreated SCLC, evidence for LCNEC remains limited to rare case reports [36]. To the best of our knowledge, this is the first report to evaluate a correlation between tarlatamab efficacy and DLL3 expression/CD8+ TILs infiltration in both DLL3‐positive and negative LCNEC cases. Interestingly, both representative cases were negative for CD8+ TILs, but the antitumor response was different. One case was DLL3 (+) which demonstrated a remarkable response to tarlatamab despite CD8+ TILs infiltration, but was ineffective for the DLL3 (−) case. Tarlatamab exerts antitumor effects by bridging DLL3‐expressing tumor cells with CD3‐expressing T‐cells. Tarlatamab could induce the recruitment of cytotoxic T‐cells to TME. Since the absence of DLL3 expression might be a negative predictive factor for DLL3‐BiTE, DLL3 (−) patients were excluded in a recent prospective study investigating novel DLL3‐BiTE therapy (obrixtamig) for NEC patients [21].

A previous report demonstrated that lung NEC with DLL3 (+) had a longer OS compared to DLL3 (−) since these patients were treated with DLL3‐BiTE [22]. These findings suggest that although DLL3 (+) lung NEC showed a poor prognosis, DLL3‐targeted therapy could improve OS. Recently, in a Phase I study investigating obrixtamig in DLL3 (+) patients, both objective response (ORR) and duration of response (DoR) were better for the DLL3‐high population (cut‐off, 50%) compared to DLL3 low [37]. DLL3 expression levels might be potential predictive biomarkers for the efficacy of DLL3‐BiTE.

This study included several limitations. First, it was a single‐center, retrospective analysis with a small sample size. Due to the rarity of LCNEC, formal a priori sample size calculation or power analysis was not conducted. Although the limited sample size may have reduced the statistical power for certain subgroup analyses, our findings provide valuable real‐world insights into this rare disease. Future validation using prospective multi‐center cohorts is warranted. Second, we did not consider clinical stage/histological subtypes (combined LCNEC or pure LCNEC) and previous treatments including ICIs in our cohort. These factors might influence survival analyses according to DLL3 status and TILs grades. Finally, an appropriate DLL3 expression threshold is still unclear to predict the efficacy of DLL3‐BiTE. We adopted a cut‐off of ≥ 1% positive tumor cells based on previous studies [14, 15, 16]. Appropriate cut‐off values should be validated in larger prospective studies.

In conclusion, DLL3 is frequently expressed in LCNEC and correlates with a poor prognosis. Furthermore, a majority of LCNEC cases are immunologically characterized as cold tumors lacking CD8+ TILs, which contributes to unfavorable clinical outcomes. Our representative cases demonstrated that tarlatamab induced a robust antitumor response even in a DLL3 (+) cold tumor. These findings suggest that DLL3‐BiTE represents a promising therapeutic strategy for DLL3 (+) LCNEC patients, potentially overcoming the suppressive TME.

## Author Contributions

**Shotaro Kaneko:** conceptualization, methodology, data curation, investigation, writing – original draft, writing – review and editing, formal analysis, project administration. **Naoki Furuya:** methodology, conceptualization, investigation, writing – original draft, writing – review and editing, data curation, formal analysis, supervision, project administration. **Yoshihisa Sugiura:** conceptualization, methodology, investigation, formal analysis, project administration, writing – review and editing. **Hajime Tsuruoka:** conceptualization, writing – review and editing, investigation. **Kazuhiro Nishiyama:** writing – review and editing, investigation. **Honoka Morita:** writing – review and editing, data curation, investigation. **Tomoki Okura:** writing – review and editing, data curation, investigation. **Hisashi Saji:** writing – review and editing, supervision. **Junki Koike:** writing – review and editing, supervision. **Nobuyuki Ohike:** writing – review and editing, supervision. **Masamichi Mineshita:** writing – review and editing, project administration.

## Funding

The authors have nothing to report.

## Ethics Statement

This study was approved by the institutional review board of St. Marianna University School of Medicine (approval no. 6652), in accordance with The Declaration of Helsinki and applicable Japanese regulations. Because this study was a retrospective analysis including historical cases spanning over a 10‐year period, obtaining written informed consent from all individual participants was not feasible. Therefore, in accordance with national ethical guidelines for retrospective clinical research, an opt‐out approach was applied. Information regarding the study and the opportunity to opt out was disclosed on our institutional website to ensure patients and their families could refuse participation.

## Conflicts of Interest

Naoki Furuya received personal honoraria from AstraZeneca, Chugai Pharmaceutical, Nippon Boehringer Ingelheim, Bristol‐Myers Squibb, Eli Lilly Japan, MSD, Pfizer Japan, Taiho Pharmaceutical, and Novartis outside the submitted work. Masamichi Mineshita has received grants from Nippon Boehringer Ingelheim, AstraZeneca, Chugai Pharmaceutical, Kyowa Kirin, Taiho Pharmaceutical, and Nippon Kayaku; and honoraria from Nippon Boehringer Ingelheim, AstraZeneca, Chugai Pharmaceutical. The other authors declare no conflicts of interest.

## Supporting information


**Figure S1:** CD8+ TIL density according to DLL3 expression. In the box‐whisker plot, upper and lower whiskers indicate 90 and 10 percentiles, respectively. The top and bottom of box indicate 75 and 20 percentiles, respectively.
**Figure S2:** Prognostic impacts of DLL3 expression and CD8+ TILs infiltration in curative treatment population (*N* = 29).
**Figure S3:** Prognostic impacts of DLL3 expression and CD8+ TILs infiltration in palliative chemotherapy population (*N* = 15).

**Table S1:** Details of systemic chemotherapy regimens by line of treatment.

## Data Availability

The data that support the findings of this study are available from the corresponding author upon reasonable request.
