# Supplementary material for: Clinicopathological Features of DLL3 Expression and T‐Cell Infiltration in LCNEC: Insights From Pathology and Clinical Response to Tarlatamab
Source: Thorac Cancer. 2026 Oct 15;17(20):e70400. doi: 10.1111/1759-7714.70400 (PMC13670004; doi:10.1111/1759-7714.70400)
Supplement: Supplementary file 1 [file TCA-17-e70400-s002.docx]

Table S1. Details of systemic chemotherapy regimens by line of treatment

|  | Number of patients |
| --- | --- |
| First-line Therapy | 21 |
| Platinum + Etoposide | 12 |
| Platinum + Etoposide + Anti-PD-L1 antibody | 6 |
| Platinum + Irinotecan | 1 |
| Nivolumab + Ipilimumab | 1 |
| Tegafur/gimeracil/oteracil (S-1) | 1 |
| Second-line Therapy | 16 |
| Amrubicin | 10 |
| Anti-PD-L1 antibody | 2 |
| Tarlatamab | 2 |
| Irinotecan | 1 |
| Tegafur/gimeracil/oteracil (S-1) | 1 |
| Third-line Therapy | 7 |
| Platinum + Etoposide | 1 |
| Platinum + nab-Paclitaxel | 2 |
| Topotecan | 1 |
| Amrubicin | 2 |
| Platinum + Irinotecan | 1 |
| Fourth-line Therapy | 4 |
| Platinum + Paclitaxel | 2 |
| Docetaxel | 1 |
| Docetaxel + Ramucirumab | 1 |
| Fifth-line Therapy | 3 |
| Nivolumab | 2 |
| Tegafur/gimeracil/oteracil (S-1) | 1 |
